# Crack Localization in Operating Rotors Based on Multivariate Higher Order Dynamic Mode Decomposition

**DOI:** 10.3390/s22166131

**Published:** 2022-08-16

**Authors:** Zhiwen Lu, Feng Li, Shancheng Cao, Rui Yuan, Yong Lv

**Affiliations:** 1 Hubei Key Laboratory of Mechanical Transmission and Manufacturing Engineering, Wuhan University of Science and Technology, Wuhan 430081, China; 2 The Key Laboratory of Metallurgical Equipment and Control of Education Ministry, Wuhan University of Science and Technology, Wuhan 430081, China; 3 School of Astronautics, Northwestern Polytechnical University, Xi’an 710072, China

**Keywords:** crack localization, rotors, nonlinear, higher-order dynamic mode decomposition

## Abstract

A novel output-only crack localization method is proposed for operating rotors based on an enhanced higher-order dynamic mode decomposition (HODMD), in which the nonlinear breathing crack-induced super-harmonic characteristic components from multiple vibration measurement points are simultaneously extracted to compose the corresponding super-harmonic transmissibility damage indexes. Firstly, the theoretical background of the HODMD is briefly reviewed. Secondly, the proposed crack localization method is dedicated which improving the HODMD for multivariate signals by casting the total least square method into standard HODMD and adaptively selecting the order parameter of Koopman approximation by optimizing the super-harmonic frequency vector. In addition, the super-harmonic characteristic components are evaluated and harnessed to derive the damage index based on super-harmonic transmissibility and fractal dimension. Finally, the proposed method is investigated and demonstrated by numerical simulations and experiments. Both numerical and experimental results show that the proposed method is powerful in realizing multi-crack localization for running rotors accurately and robustly in the case of no baseline information on intact rotors. Moreover, the interferences from commonly existing steps and misalignment can also be eliminated.

## 1. Introduction

Crack localization [1,2,3,4] in running rotors is a pretty important field in the health monitoring of rotating machines and is of great significance to assure the safe operation of critical equipment. For crack localization, high-resolution spatial information is normally a prerequisite. The spatial information could be provided by accurate baseline models or multiple measurement points. However, the former is always missed or difficult to obtain for complicated equipment. With the development of measurement and sensor technology, the latter choice is becoming available. In order to boost the accuracy and robustness of crack localization, this work develops a novel crack localization method through spatial characteristic information extraction from multiple-sensor responses for operating rotors with commonly existing interference factors and without baseline information on intact rotors.

Typical investigations have been conducted by applying mode shapes or their derivatives-based crack localization methods in stationery structures [5,6,7,8]. Local stiffness reduction of the rotor will be generated by cracks which will cause discontinuities or distortions of spatial displacement filed at crack locations. Thus, crack positions can be determined by detecting the crack-induced local differences between the damaged and undamaged modes. However, mode shapes are difficult to obtain for rotors under operating conditions. Furthermore, mode shapes could not deal well with the nonlinear rotors with fatigue breathing cracks, as they are derived based on linear theory, and the nonlinear information will be omitted. Consequently, it is not suitable to adopt the mode shape-based methods for crack localization in operating rotors.

Inspired by mode shapes, Cao et al. [9] proposed a concept called characteristic deflection shape (CDS) to localize cracks in engineering structures, which was an intrinsic spatial characteristic structure extracted by a certain rule from multiple-sensor signals [10]. Compared with the mode shape, the CDS is a more general definition, including but not limited to a mode shape or an operational deflection shape (ODS) [11], which will be more suitable for operating rotors. A kind of CDS with the kurtosis of ODS was proposed by Saravanan et al. [12] to localize cracks in operating rotors, and Babu et al. [13] defined a kind of CDS called amplitude deviation curve to reveal the crack locations in a rotor by extracting the crack-induced discontinuities. Those proposed methods typically focus on linear open cracks and are effective for serious damages in rotors and stationary structures.

While cracks in rotors are normally fatigue ones, the breathing phenomenon leads the system to be nonlinear. It will be more beneficial for crack localization if the local stiffness reduction and nonlinear features can be combined together. A breathing crack localization method was proposed by Prawin et al. [14] based on the spatial curvature derived from the high order frequency component from distributed sensors. The ratio of a super-harmonic to the fundamental harmonic at different measurement locations was explored by Broda et al. [15] for breathing crack localization. Breathing crack localization in rotors was realized by Lu et al. [16,17,18] by harnessing the breathing crack-induced super-harmonics to extract the CDS at high-order frequencies called super-harmonic CDSs. Moreover, breathing crack identification in structures was reviewed by Bovsunovsky and Surace [19]. This research shows that the CDS at a higher-order frequency will be more helpful for nonlinear crack localization.

Characteristic deflection shapes contain high-resolution spatial information required by crack localization and also avoid the difficulties of performing mode shape identification and limitations of the linear theory frame. This kind of method fulfills crack localization by detecting the distortions introduced by cracks in CDSs. Therefore, how to extract the crack position sensitive characteristic deflection shapes induced by weak cracks and avoid other interferences factors such as commonly existed steps and misalignment in rotors from multiple noise-polluted responses only deserve to be explored further.

Dynamic mode decomposition (DMD) proposed by Schmid [20] is a kind of data-driven method, which approximates the Koopman operator from the system responses only and does not rely on any prior assumption, and can extract the dominant characteristics of a nonlinear system. The key to original DMD is to compute the eigenvalue decomposition of the Koopman matrix or its similar matrix, which is the best-fit linear operator relating two neighboring snapshot matrices. Peng et al. [21] proposed a structural damage assessment method by combing the DMD with phase space embedding and evaluated the status by comparing the eigenvalues extracted from DMD between damaged and undamaged structures. Saito et al. [22] investigated the experimental modal analysis by DMD, and the results proved its feasibility to extract modal parameters by DMD, but the performance was sensitive to measurement noise. In order to ameliorate the noise-induced bias when dealing with the noise data, the total least square method was proposed by Hemati et al. [23], and the total least square DMD was adopted for bearing fault diagnosis in [24]. However, the performance of DMD will deteriorate when the snapshot vector of time-series data is smaller than the snapshot number [25], which is always the case as measurement points for vibration systems are always limited. Then, a more general extension called higher-order dynamic mode decomposition (HODMD), which extends the Koopman approximation in the original DMD to a higher-order Koopman approximation, was proposed and could overcome the problem by combining the original DMD with Takens’ theorem [26]. The HODMD method can decompose the multiple responses into single frequency components simultaneously, which could be an idea for CDS extraction. However, the noise problem and the order parameter selection are still challenging problems and require further investigations.

Aiming at extracting the crack position sensitive characteristic deflection shapes that are induced by weak cracks and avoiding other interferences factors from multiple noise-polluted responses, a novel output-only crack localization method is proposed with super-harmonic transmissibility damage index based on improved HODMD, which casts the total least square method into standard HODMD and selects order parameters adaptively for operating rotors under the interference of common existed steps and misalignment.

The major contributions of the work are as the following:

(1) A novel output-only crack localization method is proposed for operating rotors based on super-harmonic transmissibility characteristic deflection shapes derived damage index under the interference of commonly existing steps and misalignment and is validated numerically and experimentally.
(2) An improved HODMD algorithm is developed to enhance the noise-robust performance by casting the total least square method into standard HODMD and adaptively selecting key parameters by optimizing the super-harmonic frequency vector.
(3) The proposed method provides an alternative way to realize multivariate signals’ simultaneous decomposition and reconstruction, which would be very promising in many fields, such as operational modal analysis and structural health monitoring.
(4) Last but not least, it is a significant attempt to extend the application of the DMD-like method to damage identification of rotating equipment.

The remaining structure of the paper is as follows. In Section 2, the theoretical background of the HODMD is briefly reviewed first, and then the proposed crack localization method with the improved HODMD is dedicated. In Section 3, numerical investigations are performed for crack localization in operating rotors with steps and misalignment. In Section 4, experiments are carried out to validate the proposed method, and then the influence of the order parameter is discussed in Section 5. Finally, some key conclusions are summarized in Section 6.

## 2. Theory

### 2.1. HODMD

Considering x(t)∈M is the state variable of the system for *t* = 1, …, *m* + 1, which is evaluated by a vector of observables $g:M\to {\mathbb{R}}^{n}$, i.e., $g(x(t))={\left[g_{1}(x(t)),g_{2}(x(t)),\ldots g_{n}(x(t))\right]}^{T}\in {\mathbb{R}}^{n}$. For a mechanical system, g(x(t)) represents a vector of generalized coordinates at *t* instance, which corresponds to the response measured by *n* measurement points at t instance. The aim of the original DMD is to establish the relationship between snapshot g(x(t)) and its time-shifted snapshot as g(x(t+1))=Ag(x(t)), which can be expressed in terms of matrix form:

(1) Z=AY

where A is the Koopman matrix. Y and Z are the snapshot matrix and its time-shift snapshot matrix, respectively, which can be assembled as

(2) $$Y=\left[g(x(1)),g(x(2)),\ldots g(x(m))\right]\in {\mathbb{R}}^{n\times m}$$

(3) $$Z=\left[g(x(2)),g(x(2)),\ldots g(x(m+1))\right]\in {\mathbb{R}}^{n\times m}$$

Different from original DMD, HODMD relies on the higher order Koopman condition as [26].

(4) $$g(x(t+d))=A_{1}g(x(t))+A_{2}g(x(t+1))+\ldots +A_{d}g(x(t+d-1))$$

where *t* = 1, 2, …, *m* − *d*.

Let $g(x(t))=g_{t}$, the equation can be rearranged as the following:

(5) $${\bar{g}}_{t+1}=\bar{A}{\bar{g}}_{t}$$

where ${\bar{g}}_{t}={\left[g(x(t));g(x(t+1));\ldots ;g(x(t+d-1))\right]}^{T}\in {\mathbb{R}}^{dn}$ indicates the modified snapshot, and $\bar{A}$ denotes the modified Koopman matrix as

(6) $$\bar{A}=\left[\begin{array}{cccccc} 0 & \;\;\;\;\;\;I & \;\;\;\;\;\;0 & \;\;\;\;\;\;\ldots & \;\;\;\;\;\;0 & \;\;\;\;\;\;0\\ 0 & \;\;\;\;\;\;0 & \;\;\;\;\;\;I & \;\;\;\;\;\;\ldots & \;\;\;\;\;\;0 & \;\;\;\;\;\;0\\ \ldots & \;\;\;\;\;\;\ldots & \;\;\;\;\;\;\ldots & \;\;\;\;\;\;\ldots & \;\;\;\;\;\;\ldots & \;\;\;\;\;\;\;\ldots \\ 0 & \;\;\;\;\;\;0 & \;\;\;\;\;\;0 & \;\;\;\;\;\;\ldots & \;\;\;\;\;\;I & \;\;\;\;\;\;0\\ A_{1} & \;\;\;\;\;\;A_{2} & \;\;\;\;\;\;A_{3} & \;\;\;\;\;\;\ldots & \;\;\;\;\;\;A_{d-1} & \;\;\;\;\;\;A_{d} \end{array}\right]\in {\mathbb{R}}^{dn\times dn}$$

Then the higher order Koopman equation can be obtained as:

(7) $$\bar{Z}=\bar{A}\bar{Y}$$

where $\bar{Y}$ and $\bar{Z}$ represent the modified snapshot matrices which can be written as:

(8) $$\bar{Y}=\left[{\bar{g}}_{1},{\bar{g}}_{2},\ldots {\bar{g}}_{m-d}\right]\in {\mathbb{R}}^{dn\times (m-d)}$$

(9) $$\bar{Z}=\left[{\bar{g}}_{2},{\bar{g}}_{3},\ldots {\bar{g}}_{m-d+1}\right]\in {\mathbb{R}}^{dn\times (m-d)}$$

After this, the HODMD can be performed by computing the eigen-decomposition of the modified higher order Koopman matrix $\bar{A}$.

According to the definition of pseudo inverse, we have

(10) $$\bar{A}=\bar{Z}{\bar{Y}}^{†}$$

where ${}^{†}$ represents the Moore-Penrose pseudo inverse operator. The HODMD modes and eigenvalues are defined as the eigenvectors and eigenvalues of $\bar{A}$. As the dimension of $\bar{A}$ is always high, in order to boost the efficiency and reduce the effects of measurement noise, $\bar{A}$ can be further reduced according to singular value decomposition. According to the singular value decomposition method,

(11) $$\bar{Y}=U\Sigma V^{*}$$

where $U\in {\mathbb{C}}^{dn\times r}$, $\Sigma \in {\mathbb{R}}^{r\times r}$, $V\in {\mathbb{C}}^{(m-d)\times r}$, *r* indicates the rank of $\bar{Y}$ and $V^{*}$ represents the Hermitian transpose of V. Then, the pseudo inverse of $\bar{Y}$ can be expressed as

(12) $${\bar{Y}}^{†}=V\Sigma^{-1}U^{*}$$

As U contains the proper orthogonal modes, $\bar{A}$ can be reduced to its similar matrix by selecting proper rank *r* as:

(13) $$\tilde{A}=U^{*}\bar{A}U=U^{*}\left(\bar{Z}V\Sigma^{-1}U^{*}\right)U=U^{*}\bar{Z}V\Sigma^{-1}$$

Once the HODMD similar matrix $\tilde{A}$ is determined, the HODMD modes and eigenvalues can be estimated by eigen-decomposition of $\tilde{A}$.

Apart from decomposition, the original snapshots can also be reconstructed by the HODMD modes and eigenvalues as

(14) $$g_{t}=\sum_{j=1}^{N}a_{j}u_{j}e^{\left(\sigma_{j}+i\omega_{j}\right)\left(t-1\right)\Delta t}\in {\mathbb{R}}^{n}$$

where *N* implies the number of modes participating in the reconstruction process, $a_{j}$ is the *j*th mode amplitude, and $u_{j}=U_{y}q_{j}/{\left\|q_{j}\right\|}_{2}$ where $U_{y}$ is the left singular vectors of the original response matrix Y, $q_{j}$ is the *j*th eigenvector of $\tilde{A}$. The growth rates $\sigma_{j}$ and frequencies $\omega_{j}$ can be calculated by

(15) $$\sigma_{j}+i\omega_{j}=\ln\mu_{j}/\Delta t$$

where $\mu_{j}$ is the eigenvalue of $\tilde{A}$ and Δt is the sampling interval.

### 2.2. Enhanced HODMD Based Crack Localization Method

As for damaged systems, some of the eigenvectors or eigenvalues could be sensitive to the damage state, which could be useful for damage detection and identification. For cracked rotors, super-harmonics are the typical features; if the sensitive super-harmonics can be extracted, it will be helpful for crack identification. HODMD method is a kind of output-only method, and it provides the possibility to address this issue; therefore, it is chosen to investigate its feasibility and performance for crack localization in operating rotors.

However, there are still some issues required to be tackled before its application. First of all, the DMD method is sensitive to noise, and the crack signals are always noise-polluted, which will degrade the natural spatial-temporal characteristics extraction. Therefore, how to boost the robustness of the HODMD method is very important. Second, the features induced by cracks in rotors are always quite weak; how to represent the influence of cracks by a damage index is also critical. Though HODMD performs better in noise robustness as it combines the benefits of the original DMD and Taken’s theorem, it is still not enough.

In this work, the total-least-square method which has been used for noise reduction in the original DMD will be transplanted to the HODMD. Moreover, the order selection issue is tackled by designing an objective function to evaluate the identification accuracy of super-harmonic frequencies. After that, the super-harmonic components from all measurement points are extracted and reconstructed simultaneously, which are further examined to derive a damage index by general transmissibility and fractal dimension, and the derived damage index is investigated for crack localization for operating rotors. The details are in the following.

Assuming the noise matrices for $\bar{Y}$ and $\bar{Z}$ are $\Delta \bar{Y}$ and $\Delta \bar{Z}$ respectively, then the problem can be described as an optimization problem

(16) $$\underset{\bar{A},\Delta \bar{Y},\Delta \bar{Z}}{\min}{\left\|\left[\begin{array}{l} \Delta \bar{Y}\\ \Delta \bar{Z} \end{array}\right]\right\|}_{F},\;\mathrm{subject}\;\mathrm{to}\;\bar{Z}+\Delta \bar{Z}=\bar{A}\left(\bar{Y}+\Delta \bar{Y}\right)$$

where ${\left\|.\right\|}_{F}$ is the Frobenius norm. As demonstrated in [25], in the noise-corrupted condition, the total-least-square solution converged to the linear relationship without noise when the number of snapshots tended to infinity.

In order to solve the total-least-square problem, an augmented snapshot matrix is constructed

(17) $$R=\left[\begin{array}{l} \bar{Y}\\ \bar{Z} \end{array}\right]\in {\mathbb{R}}^{2dn\times (m-d)}$$

Suppose the best rank-r approximation of R is $R_{r}$. Then the total-least-square solution of $\bar{A}$, denoting as ${\bar{A}}_{T}$ can be obtained according to

(18) $$\hat{Z}={\bar{A}}_{T}\hat{Y}$$

where $\hat{Z}=\bar{Z}P_{r}\in {\mathbb{R}}^{dn\times (m-d)}$, $\hat{Y}=\bar{Y}P_{r}\in {\mathbb{R}}^{dn\times (m-d)}$ are the de-noise matrices respectively, and $P_{r}$ is the projection onto the range of $R_{r}$_._

$P_{r}$ can be obtained by applying SVD to R as

(19) $$R=\bar{U}\bar{\Sigma }{\bar{V}}^{*}$$

then $P_{r}={\bar{V}}_{r}{\bar{V}}_{r}{}^{*}\in {\mathbb{R}}^{\left(m-d)\times (m-d\right)}$,where ${\bar{V}}_{r}\in {\mathbb{C}}^{(m-d)\times r}$ is the first *r* columns of $\bar{V}$.

As the dimensions of $\hat{Z}$ and $\hat{Y}$ are relatively high, dimension reduction is required. We have

(20) $${\hat{Y}}_{dn\times (m-d)}={\hat{U}}_{dn\times N}{\hat{\Sigma }}_{N\times N}{\hat{V}}^{*}{}_{N\times (m-d)}$$

where $\hat{\Sigma }$ contains *N* retained singular values $\lambda_{1},\lambda_{2},\ldots ,\lambda_{N}$, the number satisfies $\frac{\lambda_{N+1}^{2}+\ldots +\lambda_{h}^{2}}{\lambda_{1}^{2}+\ldots +\lambda_{h}^{2}}\leq \varepsilon$, with h=min{d,m−d}, where ε can be set manually. Then the reduced HODMD matrix ${\tilde{A}}_{T}$ is determined by

(21) $${\tilde{A}}_{T}={\hat{U}}^{*}{\bar{A}}_{T}\hat{U}={\hat{U}}^{*}\left(\hat{Z}\hat{V}{\hat{\Sigma }}^{-1}U^{*}\right)\hat{U}={\hat{U}}^{*}{}_{N\times dn}{\hat{Z}}_{dn\times (m-d)}{\hat{V}}_{(m-d)\times N}{\hat{\Sigma }}^{-1}{}_{N\times N}$$

Then HODMD modes and eigenvalues can be obtained by eigen-decomposition of ${\tilde{A}}_{T}$.

In the HODMD, order parameter *d* is important and usually selected manually, which will not be user-friendly. Therefore, in this work, the determination of *d* is solved by an objective optimization. The objective function is constructed by the first p order frequencies obtained by HODMD. Assume that the first *p* order frequencies are $W=\left[\omega_{1},\omega_{2},....,\omega_{p}\right]$, and the theoretical frequencies are $W_{o}=\left[\omega_{o},2\omega_{o},....,p\omega_{o}\right]$, where $\omega_{o}$ is the rotating speed during the measurement. Then *d* can be determined by the following optimization problem:

(22) $$\underset{d}{\min}{\left\|W-W_{O}\right\|}_{\infty }/{\left\|W_{O}\right\|}_{\infty }$$

After the determination of optimized *d*, the snapshots $\bar{g}(x(t))$ can be reconstructed by the HODMD modes obtained by the optimal parameters. If the selected HODMD modes for reconstruction correspond to the super-harmonic frequencies, then the corresponding super-harmonic components can be obtained. From Equation (14), we have

(23) $${\tilde{g}}_{t}^{k}=\sum_{j}a_{j}u_{j}\mu_{j}^{t-1}\in {\mathbb{R}}^{n}$$

where *k* is the order number of the super-harmonic component. Then the extracted super-harmonic matrix can be obtained by

(24) $${\tilde{Y}}^{k}=\left[{\tilde{g}}_{1}^{k},{\tilde{g}}_{2}^{k},\ldots ,{\tilde{g}}_{m}^{k}\right]=\left[y_{1}^{k};y_{2}^{k};\ldots ;y_{n}^{k}\right]\in {\mathbb{R}}^{n\times m}$$

where $y_{i}^{k}$ is the *i*th row of ${\tilde{Y}}^{k}$, which corresponds to the super-harmonic component extracted from the response of the *i*th measurement point.

As transmissibility is sensitive to the local variation in nature, it gives the advantage for damage detection [27,28]. As crack-induced nonlinearity is local, the closer the measurement point to the crack, the more influence will be the crack on the transmissibility between the current measurement point and a fixed reference measurement point. In this case, the cracks will lead to distortions near the crack positions if all the transmissibility from all measurement points forms into a curve. In contrast, no distortions will appear when there are no cracks, and the curve will be smooth. In order to indicate the crack positions more clearly, a local complexity evaluation method called fractal dimension will be proposed to derive a damage index to amplify the weak distortions. The derivation of the damage index is explained as the following.

According to the definition of transmissibility,

(25) $$T_{ab}^{k}(\omega )=\frac{\left|G_{ab}^{k}(i\omega )\right|}{\left|G_{bb}^{k}(i\omega )\right|}$$

where *a* and *b* are any two measurement point positions, and $G_{ab}^{k}(i\omega )$$G_{bb}^{k}(i\omega )$ are the corresponding cross- and auto-power spectra, respectively.

Suppose that the *k*th order super-harmonic transmissibility vector of all measurement points to the first reference point is $T^{K}=[T_{11}^{k},T_{21}^{k},\ldots ,T_{n1}^{k}]$ and its corresponding curve called transmissibility characteristic deflection shape (TCDS) is composed of the *n* points $\left(O_{1},\ldots ,O_{n}\right)$. A sliding window with a width of w and a sliding step of one point is applied to truncate the kth order super-harmonic TCDS curve to show its local complexity. Then the fractal dimension [29] damage index of point *j* can be expressed as:

(26) $$DI\left(j\right)=\frac{\mathrm{lg}\left(w-1\right)}{\mathrm{lg}(w-1)+\mathrm{lg}(s\left(j\right)/L\left(j\right))}$$

(27) $$s\left(j\right)=\max_{j\leq q\leq j+w-1}\mathrm{dist}(O_{j},O_{q})$$

(28) $$L\left(j\right)=\sum_{q=j}^{j+w-2}\mathrm{dist}(O_{q},O_{q+1})$$

where dist(⋅,⋅) represents the distance between two points. The positions of peaks of DI indicate the positions of cracks.

**Figure 1:**
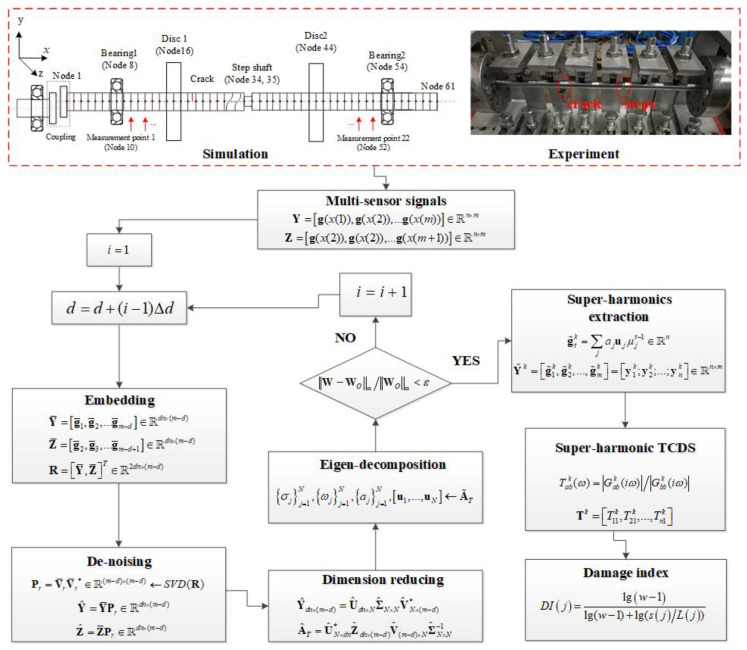
The flowchart of the proposed crack localization method.

To sum up, the general procedure of the proposed crack localization method is described in Figure 1.

## 3. Numerical Investigation

**Figure 2:**
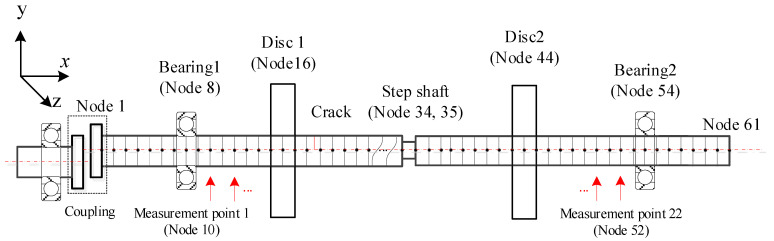
Schematic diagram of the numerical stepped rotor with cracks and misalignment.

The numerical rotor model shown in Figure 2 is used to investigate the performance of the proposed method. In the model, the shaft is discretized equivalently by two-node twelve degrees of freedom (DOFs) Timoshenko beam elements, where each node contains three translational and three rotational DOFs. Each disc is considered a rigid body modelled by six DOFs lumped inertias at the corresponding node. The bearings are represented by isotropic spring-damping systems. A shaft with a reduced diameter is used to model the step shaft. Transverse cracks with breathing phenomenon are considered and modelled by linear fracture mechanics with strain energy release rate theory and the Crack Closure Line Position (CCLP) method [30]. In order to validate the performance of the proposed method to eliminate the interference of commonly existing super-harmonic components, sample parallel misalignment is considered by external excitation with two times rotating frequency [31]. Torsional and axial DOFs of the rotor in the driving end are fixed. Finally, the motion equation of the rotor system is established by assembling all element matrices as:

(29) $$M\ddot{q}+\left(D+\Omega D_{g}\right)\dot{q}+K\left(t\right)q=F_{u}+F_{g}+F_{c}$$

where q is the displacement vector of all nodes, M is the system mass matrix, D is the system Rayleigh damping matrix, Ω is the rotating frequency, $D_{g}$ is the system gyroscopic matrix, K(t) is the system stiffness matrix, $F_{u}$ is the unbalance excitation force vector, $F_{g}$ is the gravitational excitation force vector, $F_{c}$ is the external excitation force vector which is the misalignment excitation vector in this work.

From the motion equation, one can see the system stiffness matrix K(t) introduced by breathing cracks is time-varying and state-dependent, which makes the system nonlinear and generates super-harmonic components, and the responses are obtained by solving the equation via the Newmark-beta method [32] with a 5000 Hz sampling frequency. The details of the model, except the misalignment information, can be found in the authors’ previous work [16,17]. The excitations in the vertical and horizontal DOFs induced by coupling misalignment in $F_{c}$ can be expressed as

(30) $$\left\{ \begin{array}{l} F_{\mathrm{cy}}=m_{c}\Delta y\Omega^{2}\cos(2\Omega )\\ F_{\mathrm{cz}}=m_{c}\Delta y\Omega^{2}\sin(2\Omega ) \end{array}\right.$$

where $m_{c}$ is the mass of coupling housing, Δy is the parallel misalignment amount and Ω is the rotating frequency.

In order to operate the numerical experiment, 22 virtual ‘vertical displacement sensors’ are arranged every two nodes equivalently between the two bearings from node 10 to node 52, corresponding to the measurement points 1 to 22 as shown in Figure 2, and the ‘measured’ signals from the measurement points are the vertical displacement responses of the corresponding nodes, and then the obtained vertical displacement responses are used simultaneously to extract super-harmonic components and derive the damage index by the proposed HODMD method following the flow chart shown in Figure 1. In order to investigate the performance of the proposed method, typically stepped rotors with single or multiple cracks and parallel misalignment are considered. The stepped shaft is set between measurement points 12–13, with a diameter of 0.8*D*, where *D* is the diameter of the shaft without steps which is 10 mm in this work. The crack and interference parameters of the numerical rotors are tabulated in Table 1.

### 3.1. Localization Results for Simulations

**Figure 3:**
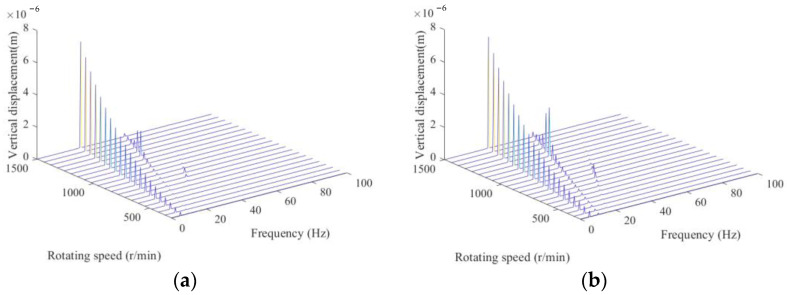
Typical waterfall spectrum of numerical rotors: (**a**) case one; (**b**) case two.

To investigate the performance of the proposed crack localization method, responses from numerical rotors in Table 1 are obtained and used for crack localization. Typical waterfall spectrum is illustrated in Figure 3.

From Figure 3, one can see 1x, 2x and 3x (x corresponding to the rotating frequency) components are generated in the rotor with breathing cracks, which shows obvious super-harmonic characteristics, and the super-harmonics can be utilized to detect the existence of cracks just by only one measurement point. However, it cannot determine the number and position of cracks without baseline models. In order to solve this problem, we propose to integrate multiple measurement points, and based on the principle that the closer is the crack to the measurement point, the more influence will be the stiffness reduction and nonlinearity induced by cracks on the responses.

The proposed HODMD method is used to simultaneously extract the 1x, 2x and 3x components from all 22 measurement points, and the corresponding transmissibility characteristic deflection shapes (TCDSs) are obtained, and fractal dimension damage index of local TCDS are computed for every point to evaluate the influence of cracks, and finally realizes crack localization.

The order parameter *d* in Equation (4) of HODMD is very important for accurate harmonic components extraction in noisy condition. To determine the parameter *d*, optimisation is performed by find the optimal *d* to minimize the difference between extraction super-harmonic frequency vector and theoretical super-harmonic frequency vector with Equation (22).

**Figure 4:**
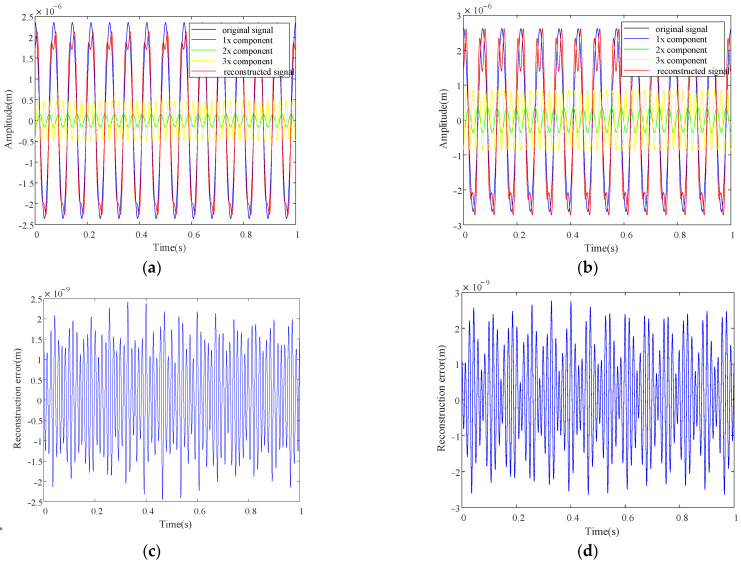
Extracted harmonic components based on HODMD for numerical rotors: (**a**) case one; (**b**) case two; (**c**) reconstruction error of case one; (**d**) reconstruction error of case two.

Figure 4 shows the extracted harmonic components for case one and case two at 840 r/min (which equals to the 1/3 critical speed of the rotor system) with *d* = 400. From the results one can see that the original signals are decomposed into single frequency components by HODMD, and the single frequency components can reconstruct the original signals accurately which makes the original and reconstructed curves almost overlap each other, and the reconstruction errors by the first three harmonics are shown in Figure 4c, d for case one and case two respectively. The magnitude of reconstruction errors is 10^−9^ m compared to 10^−6^ m of the original signal, which shows the accuracy of the decomposition by the proposed method, and the frequency components in the error diagrams are mainly higher frequencies, as only the first three order components are used for reconstruction. Moreover, the decomposition for all measurement points is simultaneously, which makes the HODMD well suit for the characteristic deflection shapes extraction.

**Figure 5:**
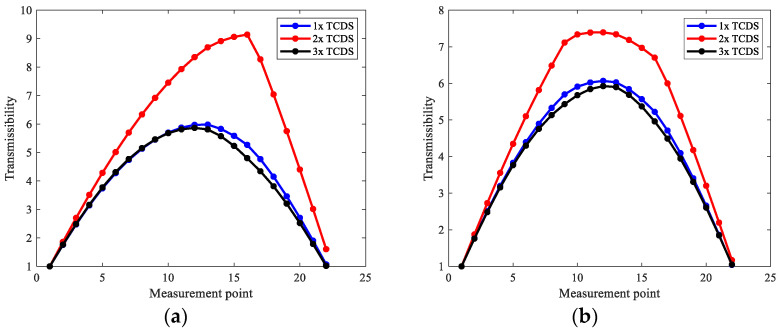
TCDS for the first three harmonics of numerical rotors: (**a**) case one; (**b**) case two.

**Figure 6:**
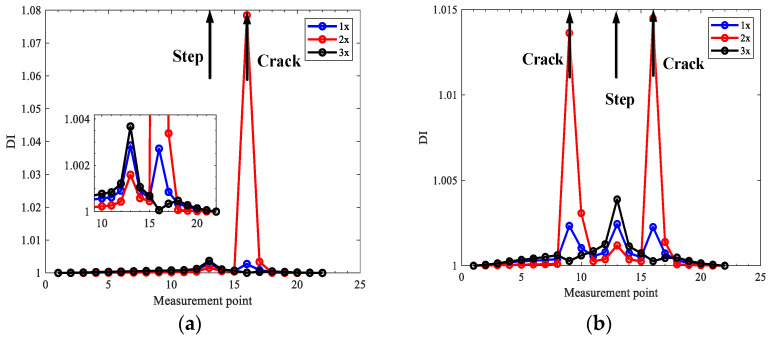
Damage index of the first three TCDSs for numerical rotors: (**a**) case one; (**b**) case two.

Based on the extracted harmonic components, the transmissibility between all measurement points and the first reference point for 1x, 2x, and 3x components, respectively, is obtained, which composes the first three TCDSs shown in Figure 5, and the corresponding damage indexes are computed shown in Figure 6.

From Figure 5, one can see obvious distortions appear in the measurement points near cracks, especially for 2x TCDSs, in both case one and case two. However, this is not clear to localize cracks. However, the peaks clearly indicate the crack locations by the derived damage index in Figure 6. From Figure 6, one can see that the damage index from 2x TCDS can not only locate the cracks but also reduce the interference of steps which shows a small peak compared to the peak of the crack. While, the interference of steps on the damage index from 1x and 3x TCDSs cannot be omitted, which will mislead the crack localization.

### 3.2. Effects of Rotating Speed

**Figure 7:**
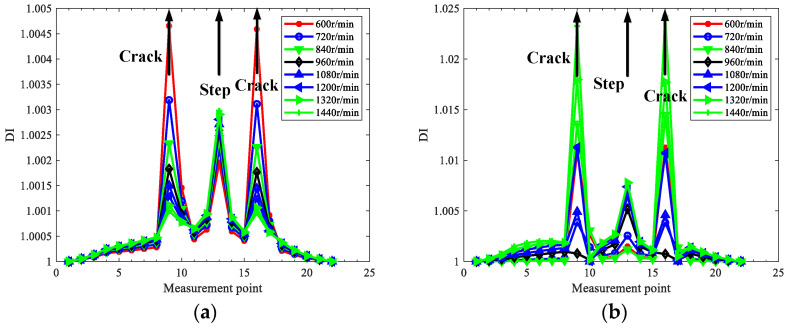

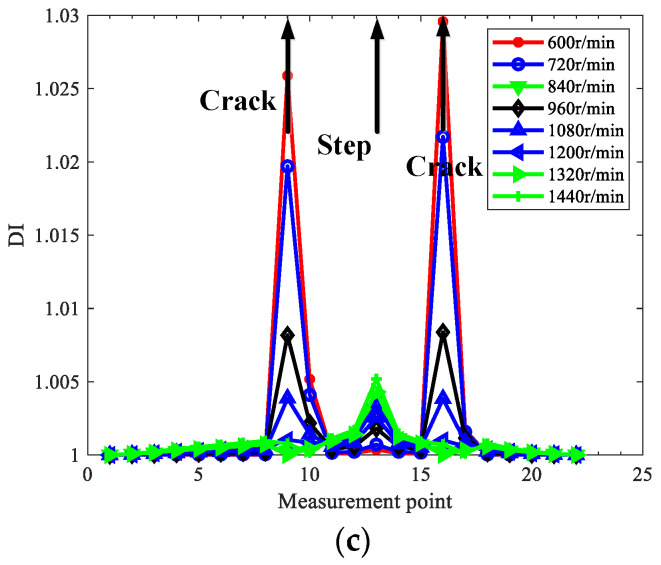
Localization results in case two under various rotating speed using TCDSs: (**a**) 1x; (**b**) 2x; (**c**) 3x.

As the rotating speed relates to harmonics in the responses, in order to explore how to eliminate the interference of steps, the influence of rotating speed is investigated for the rotor in case two without losing generality. The effects of rotating speed on the localization results of the rotor in case two using 1x, 2x, and 3x TCDSs are depicted in Figure 7.

From Figure 7, one can see the rotating speed has a great influence on localization results, and the interference of steps can be eliminated by the proper selection of rotating speed. In detail, the higher the rotating speed, the more interference in the steps; hence, from the point of reducing step interference, low speed could be helpful for crack localization for stepped rotors. The interference of steps in 1x TCDS is always obvious and cannot be eliminated, regardless of the rotating speeds; however, 2x or 3x TCDS with relatively low speed can exclude the interference of steps.

As to accurately locate cracks and reduce the step interference, the extracted TCDS should contain more nonlinear contribution from breathing cracks, and the contribution from the linear modes should be as little as possible. For 1x TCDS, the main contribution is always the first linear mode, which cannot eliminate the step interference. In contrast, for 2x or 3x TCDSs under lower speeds, the corresponding frequencies are away from the critical frequency, which makes little contribution to the linear mode, and leads to better performance in step interference reduction. Hence, the main principle to set rotating speed is to make the 2x or 3x frequency away from the critical speed, and it is more preferred to use 2x TCDS among 1x–3x TCDSs to perform crack localization for stepped rotors, as it is more possible to satisfy the rotating speed demand to eliminate the interference of steps. In the following, only the 2x TCDS will be investigated for crack localization.

### 3.3. Crack Localization for Misaligned Rotors

**Figure 8:**
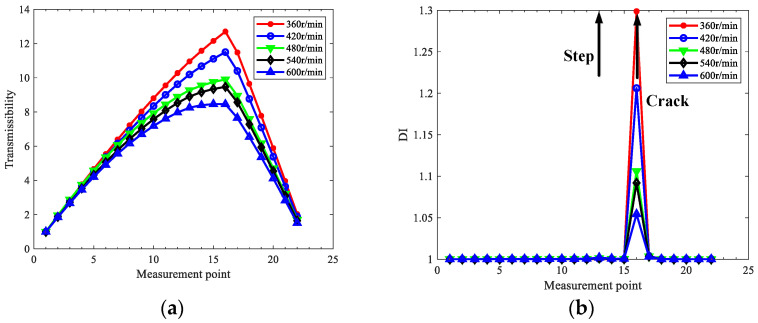
Extracted 2x TCDS and its corresponding crack localization result for case three with misalignment: (**a**) 2x TCDSs; (**b**) localization results.

**Figure 9:**
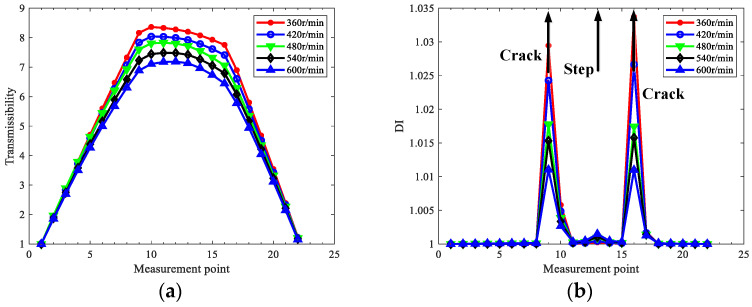
Extracted 2x TCDS and its corresponding crack localization result for case four with misalignment: (**a**) 2x TCDSs; (**b**) localization results.

Super-harmonic components will be generated by commonly existing coupling misalignment in rotors, which will affect crack detection. Therefore, cases three and four with parallel misalignment are considered. The misalignment parameters $m_{c}$ and Δy in Equation (30) are set as 0.1 Kg and 1 mm, respectively, and 2x TCDSs are extracted by the proposed method to localize the cracks under the 2x component interference by misalignment. Figure 8 and Figure 9 show the extracted 2x TCDS and its corresponding damage index for case three and case four, respectively. The rotating speeds are considered from 360 r/min to 600 r/min to reduce the interference of steps, and the order of HODMD is the same as the above investigation with *d* = 400.

From Figure 8 and Figure 9, the cracks are localized accurately, and both the interference of misalignment and steps can be excluded, and the performance is better with low speeds than with high ones.

### 3.4. Effects of Signal Noise

**Figure 10:**
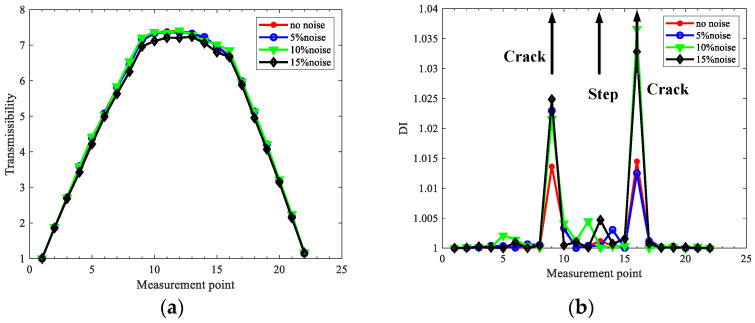
Extracted 2x TCDSs and its corresponding crack localization results for case two under various level of noise: (**a**) 2x TCDSs; (**b**) localization results.

Noise in measurement signals cannot be avoided, and it is always heavy in real applications. In order to explore the robustness of the proposed method to noise, rotors in case two under different noise levels are investigated for crack localization by using 2x TCDSs. The noise-polluted response $y_{N}$ is obtained by adding Gaussian white noise. In order to compare the results for rotors in case two without noise above, the rotating speed is selected at 840 r/min, and the order parameter *d* = 400; the extracted 2x TCDSs and localization results under different levels of noise are shown in Figure 10.

As shown in Figure 10, the peaks indicating cracks are obvious at the crack locations under noise levels from 0 to 15%, and interference from the steps can be neglected. The result indicates the good noise robustness of the proposed method. It should be noted that optimizing the order parameter *d* further would obtain better noise robustness of the proposed method, which will be discussed in Section 5.

## 4. Experimental Validation

**Figure 11:**
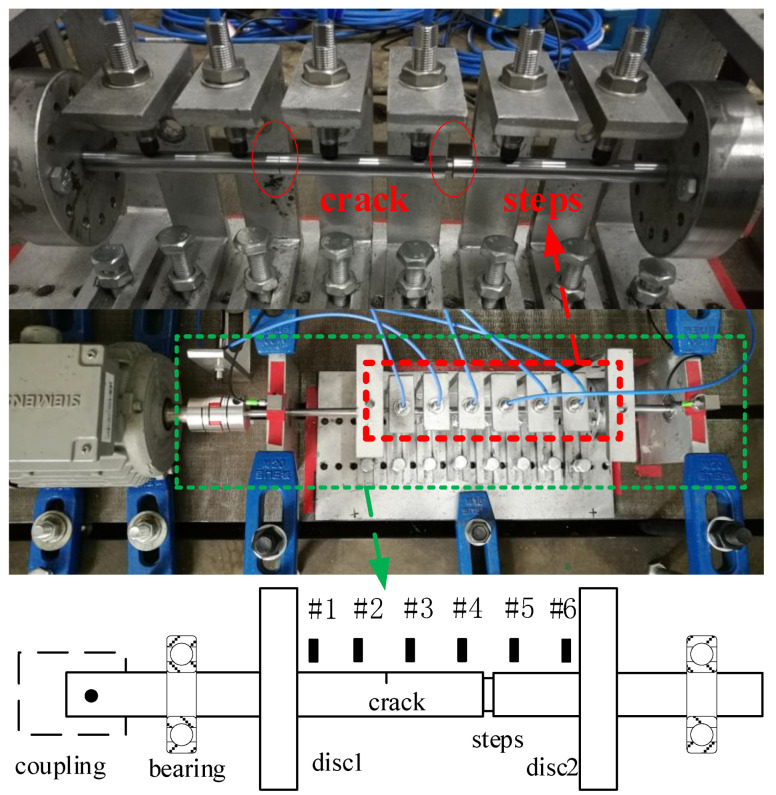
Motor-driven two-disc rotor-bearing test rig for crack localization.

The proposed crack localization method will be validated by lab experiments in this section. The motor-driven two-disc rotor-bearing test rig is graphed in Figure 11. Cracks are manufactured by wire cutting, and the step shaft is simulated by a ring slot. Unbalance mass is added by a bolt in the disc, and residual misalignment is generated by no strict alignment of the rotor. Multiple-sensor responses are obtained by arranging six eddy current vertical displacement sensors along the shaft with a 5000 Hz sampling frequency. The rotating speeds are set as 600 r/min and 720 r/min to make the 2x frequency component away from critical speed. Detailed configuration parameters of the cracks and steps in the under-test rotors are provided in Table 2.

**Figure 12:**
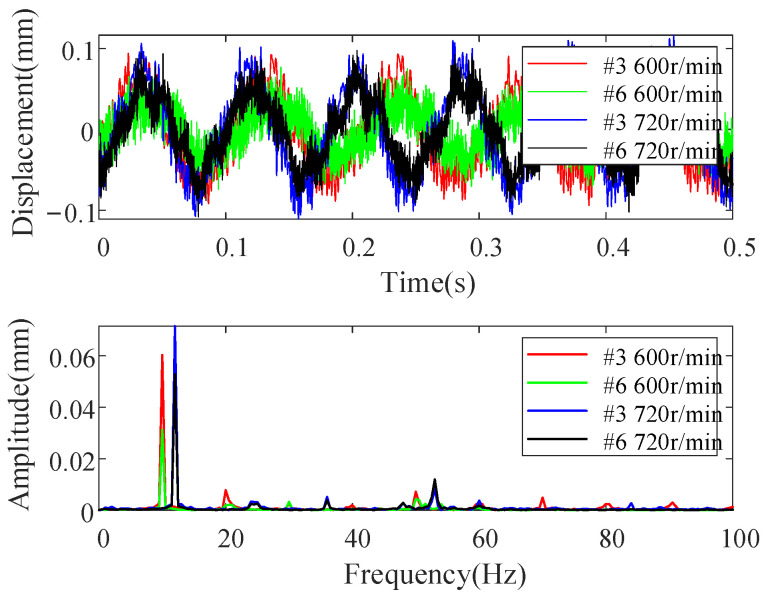
Typical measured responses in case three from measurement points #3 and #6 at rotating speed of 600 r/min and 720 r/min.

**Figure 13:**
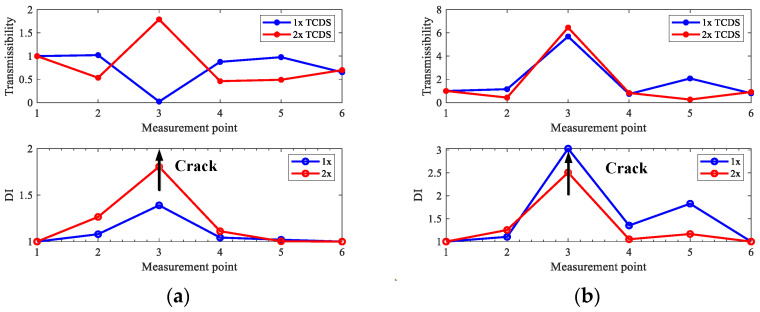
Crack localization results by the proposed method for experimental rotors in case one: (**a**) 600 r/min, *d* = 500; (**b**) 720 r/min, *d* = 500.

**Figure 14:**
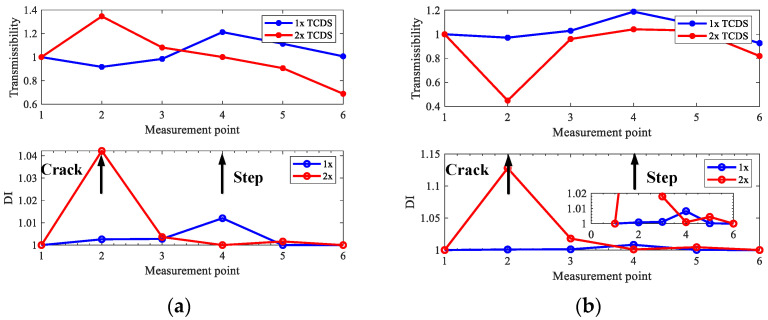
Crack localization results by the proposed method for experimental rotors in case two: (**a**) 600 r/min, *d* = 200; (**b**) 720 r/min, *d* = 600.

**Figure 15:**
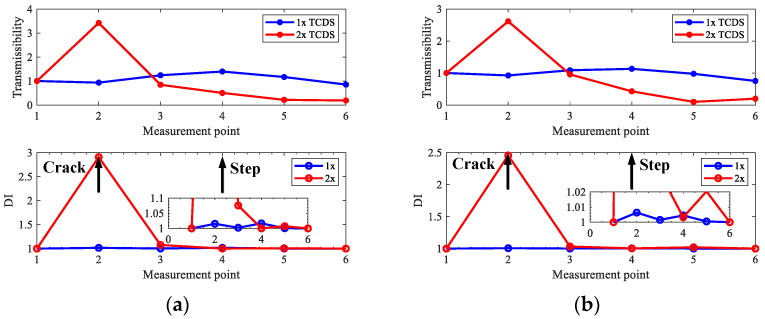
Crack localization results by the proposed method for experimental rotors in case three: (**a**) 600 r/min, *d* = 550; (**b**) 720 r/min, *d* = 400.

Figure 12 shows the typical vibration responses and the corresponding spectrums measured from sensors #3 and #6 for the rotor in case three. It can be seen from Figure 12 that the measured responses are polluted by heavy noise. In order to make the figure clearer, only frequencies lower than 100 Hz are displayed in Figure 12, and the main frequencies in the responses are the harmonics of rotating frequency induced by unbalance, misalignment, and cracks. Therefore, the experimental rotors are multiple fault ones to some degree, in addition to the steps, which makes the crack localization more challenging, and it will be convinced if the proposed method can perform well in this condition. To be consistent with the above numerical studies, only 1x and 2x TCDSs are considered to validate the proposed method. Crack localization results for the three rotor cases in Table 2 are shown in Figure 13, Figure 14 and Figure 15.

From the results for the rotor in case one without steps in Figure 13, one can see that both 1x and 2x TCDSs can detect the crack position correctly at the set rotating speeds, and what can be seen from Figure 14 and Figure 15, the cracks on the rotors in case two and three can be located distinctly by the 2x TCDSs and the interferences of steps can be eliminated. However, the steps cannot be excluded when using 1x TCDSs. Furthermore, as the misalignment exists in all experimental cases, therefore the interferences of misalignment can also be circumvented, which validates the good performance of the proposed crack localization method.

## 5. Discussion

**Figure 16:**
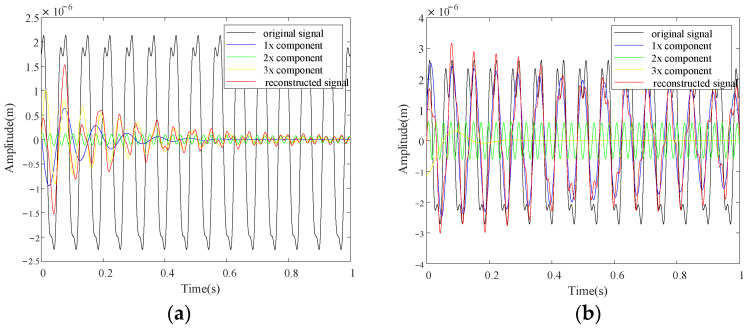
Extracted harmonic components based on HODMD when *d* = 1 for numerical rotors at 840 r/min: (**a**) case one; (**b**) case two.

From the above numerical and experimental investigation, it is validated that the proposed method can realize crack localization for rotors in operation with steps and misalignment, and the rotating speed and order parameter selection are very important. The effect of rotating speed has been studied in Section 3. Here, we want to discuss the effect of the order parameter in HODMD. Figure 16 shows the harmonics component extraction result for simulation cases one and two with *d* = 1, which means the HODMD method reduces to the DMD method. All the condition in Figure 16 is the same as that in Figure 4 except the value of order parameter *d*.

From Figure 16 and compared with Figure 4, one can see the extracted harmonic components are not stable. Moreover, the difference between the original signals and the reconstructed signals is quite large, which means the decomposition results are not accurate. The frequencies of the 1x, 2x, and 3x components for case one are 9.94 Hz, 16.84 Hz, and 40.27 Hz, and those for case two are 5.37 Hz, 14.41 Hz, and 41.51 Hz, which are all deviated from the accurate ones 14 Hz, 28 Hz, and 42 Hz. Hence, the decomposition with *d* = 1 is a failure, which validates the necessity to apply the HODMD.

**Figure 17:**
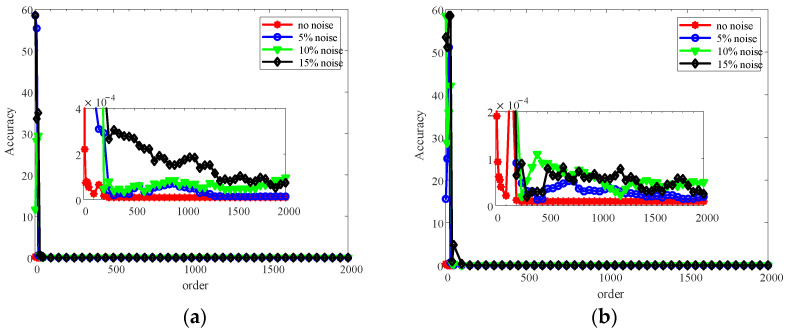
Evolution of decomposition accuracy of HODMD with various orders for numerical rotors at 840 r/min: (**a**) case one; (**b**) case two.

Then, how will the order parameter influence the decomposition results? In order to discuss this question, various values of *d* are explored, and Figure 17 shows the relationship between the value of *d* and the decomposition accuracy evaluated by the infinite norm between the extracted harmonic frequencies and theoretical ones with Equation (22) for cases one and two with various level of noise.

It can be concluded from Figure 17 that the higher order will generate higher accuracy on the whole, and the higher noise level will lead to more fluctuation of the accuracy with orders. However, the higher order will lead to heavier computation complexity. Therefore, in order to balance the accuracy and efficiency, it is required to make balance and optimize the order parameter.

## 6. Conclusions

A novel crack localization method is proposed for operating rotors based on the improved multivariate higher-order dynamic mode decomposition (HODMD) and the super-harmonic transmissibility characteristic defection shapes (TCDSs) with fractal dimension damage index by using the nonlinearity and stiffness reduction induced by cracks. The main conclusions are as the following:

(1) The proposed crack localization method is available for operating rotors with multiple cracks and has been validated by numerical and experimental investigation, which is output-only, baseline-free, and noise-robust, and the interferences from the commonly existing steps and misalignment in rotors can be eliminated.
(2) By casting the total least square method into standard HODMD and adaptively selecting the order parameter of Koopman approximation by optimizing the super-harmonic frequency vector, the improved HODMD method can deal with the multivariate noise-contaminated signals from multiple measurement points simultaneously. In view of the characteristics of the method, it provides an alternative way for multivariate signal processing.
(3) Proper selection of rotating speed for crack localization can help to eliminate the interferences of steps in rotors. The main selection principle is to make super-harmonic components away from the critical speed, and lower speeds perform better.
(4) The order parameter in the proposed method is important for the accuracy of decomposition. Higher orders seem better for accuracy, but not absolutely, and the efficiency will be lower; hence, the optimal order is demanded.

Though the good performance of the proposed method in numerical and experimental cases, the main limitation is the multiple measurement points requirement for real applications. However, the number of measurement points will just affect the resolution of localization results which is determined by the resolution demanded by users, the more the measurement point, the higher localization resolution will be, and with the development of non-contact and embedding measurement technology, this limitation could be overcome in the near future. In addition, though the proposed method is promising in real applications, more experimental investigation and application research should be further carried out to strengthen the applicability of the method.

## Figures and Tables

**Table 1:** Crack and interference parameters of the numerical rotors.

Simulation Cases	Crack 1		Crack 2		Stepped Shaft	Misalignment
	Position (Measurement Point)	Depth (mm)	Position (Measurement Point)	Depth (mm)	Position (Measurement Point)	
1	15–16	1.5	--	--	12–13	--
2	8–9	1.5	15–16	1.5	12–13	--
3	15–16	1.5	--	--	12–13	Parallel
4	8–9	1.5	15–16	1.5	12–13	Parallel

**Table 2:** Cases for experimental rotors.

Case	Crack Location (Measurement Point)	Crack Depth (mm)	Step Location (Measurement Point)
1	3–4	1.57	--
2	2–3	1.54	4–5
3	2–3	3.29	4–5

## Data Availability

Data set available on request to corresponding authors.
